# Incidental found tuberculous peritonitis during hernioplasty

**DOI:** 10.1093/jscr/rjaf328

**Published:** 2025-05-22

**Authors:** Yu-Hsin Tu, Wei-Chang Huang, Wan-Tzu Lin, Jing Tong Fu, Sheng-Chun Hung

**Affiliations:** Department of Urology, Taichung Veterans General Hospital, 1650 Taiwan Boulevard Sect. 4, Taichung 407219, Taiwan; Department of Education, Taichung Veterans General Hospital, 1650 Taiwan Boulevard Sect. 4, Taichung 407219, Taiwan; Division of Chest Medicine, Department of Internal Medicine, Taichung Veterans General Hospital, 1650 Taiwan Boulevard Sect. 4, Taichung 407219, Taiwan; Department of Medical Technology, Jen-Teh Junior College of Medicine, Nursing and Management, NO,79-9 Sha-Luen Hu Xi-Zhou Li Hou-Loung Town, Miaoli 350, Taiwan; Department of Life Sciences, National Chung-Hsing University, 145 Xingda Rd., South Dist., Taichung City 402202, Taiwan; Department of Industrial Engineering and Enterprise Information, Tunghai University, No. 1727, Sec. 4, Taiwan Boulevard, Xitun District, Taichung 407224, Taiwan; Division of Gastroenterology and Hepatology, Department of Internal Medicine, Taichung Veterans General Hospital, 1650 Taiwan Boulevard Sect. 4, Taichung 407219, Taiwan; Post-Baccalaureate Medicine, National Chung Hsing University, 145 Xingda Rd., South Dist., Taichung City 402202, Taiwan; Department of Pathology and Laboratory Medicine, Taichung Veterans General Hospital, 1650 Taiwan Boulevard Sect. 4, Taichung 407219, Taiwan; Department of Urology, Taichung Veterans General Hospital, 1650 Taiwan Boulevard Sect. 4, Taichung 407219, Taiwan; Post-Baccalaureate Medicine, National Chung Hsing University, 145 Xingda Rd., South Dist., Taichung City 402202, Taiwan; Institute of Medicine, Chung Shan Medical University, No. 110, Section 1, Jianguo North Road, Taichung 402, Taiwan

**Keywords:** hernia, peritonitis, abdominal tuberculosis, peritoneal tuberculosis

## Abstract

We report the case of a 71-year-old male with palpable and painful left inguinal mass who underwent open hernioplasty. During the operation, ~700 ml of ascitic fluid and multiple nodules on the peritoneum were observed upon incision of the hernia sac. Culture and pathological examination confirmed a diagnosis of peritoneal tuberculosis (TB). Abdominal TB is rarely presented as a hernia. We review ten cases of abdominal TB associated with abdominal hernias and discuss surgical techniques and further management options.

## Introduction

Abdominal tuberculosis (TB) accounts for ~5% of all TB cases worldwide [1]. Peritoneal TB is estimated to represent more than two-thirds of these cases, while less common forms include intestinal, luminal, and abdominal-nodal TB [2, 3]. Abdominal TB is notable for its varied clinical manifestations, which may present as acute abdomen, intestinal perforation or obstruction, or chronic abdominal pain. Inguinal hernia is a common condition characterized by the protrusion of intra-abdominal contents, such as the intestines, through a weak spot in the abdominal wall, specifically in the inguinal region. Clinically, patients may present with a noticeable bulge in the groin area, which becomes more prominent during activities such as lifting or coughing. Surgical intervention is the primary treatment to prevent complications like bowel obstruction or strangulation. To date, only 10 cases of abdominal TB associated with abdominal hernias have been reported in the English-language literature [4–13], with two involving a pediatric patient [8, 12]. Here, we report an unusual case of an inguinal hernia in an adult patient with peritoneal TB.

## Case report

A 71-year-old male presented with a one-month history of a palpable and painful left inguinal mass, accompanied by a dragging sensation. His medical history included chronic obstructive pulmonary disease, hypertension, type II diabetes, and hyperlipidemia. He had no history of TB. The pain worsened with walking and was alleviated by rest. Physical examination revealed a left inguinal hernia, and the patient was scheduled for herniorrhaphy.

During surgery, an indirect hernia sac was identified originating from the internal inguinal ring. Upon rupture of the hernia sac, ~700 ml of massive ascitic fluid was encountered. Additionally, multiple white nodules were observed on the peritoneum. Cytological and pathological samples were obtained. Given the high risk of infection, a mesh was not utilized, and the Bassini repair technique was employed instead.

**Figure 1:**
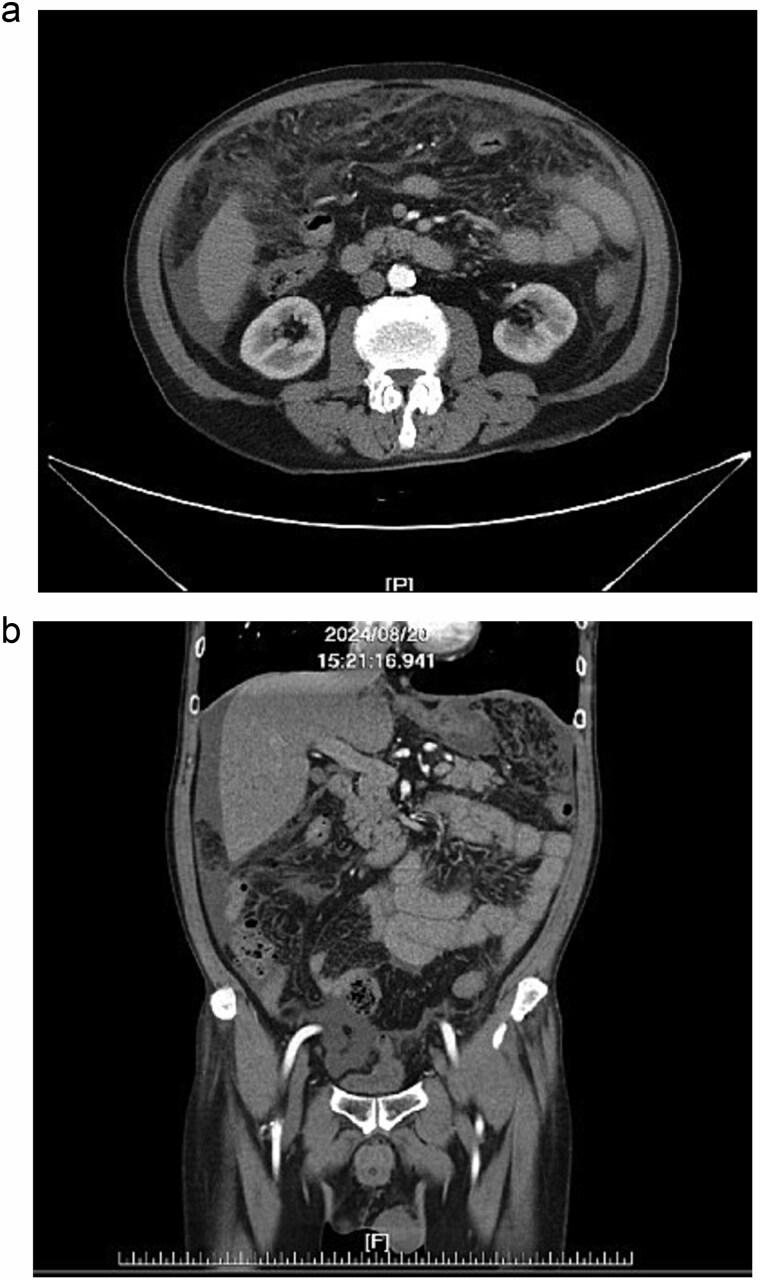
(a, b) Abdominal CT, peritoneal thickening with multiple miliary nodules and omental cake with ascites.

**Figure 2:**
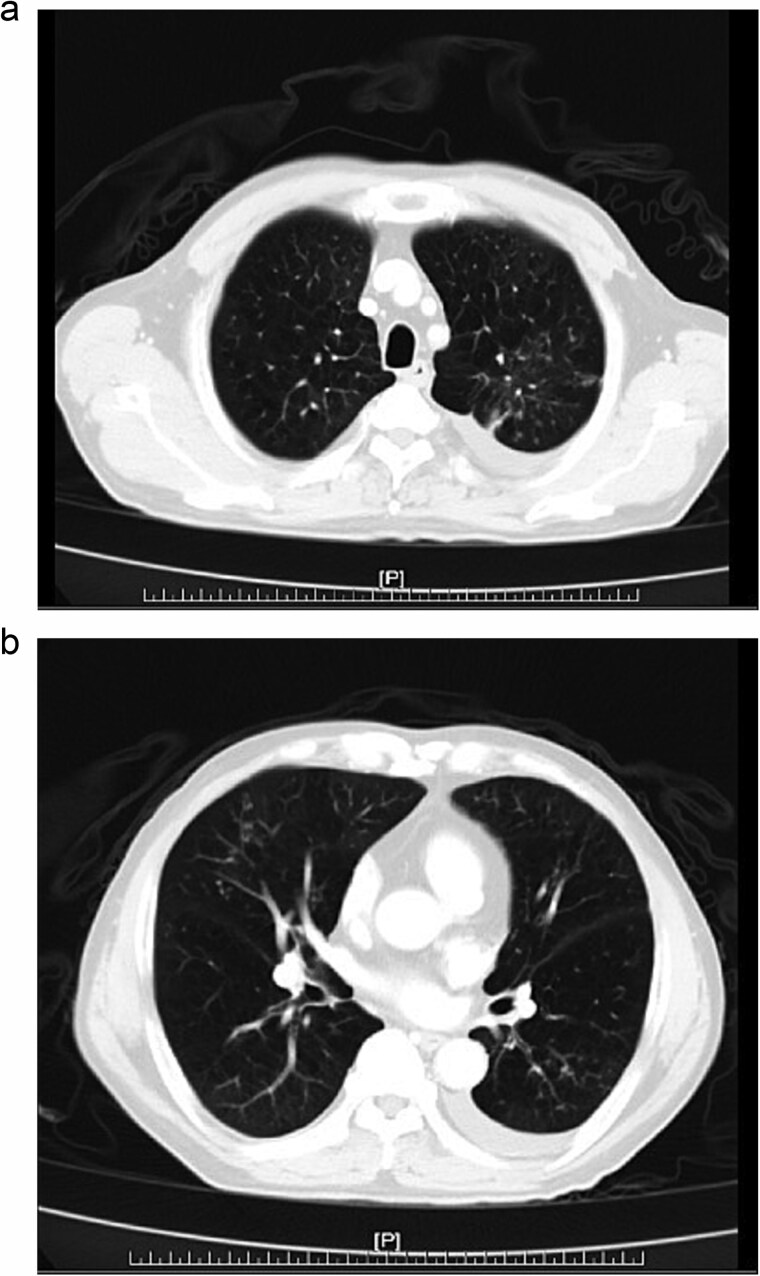
(a, b) Chest CT, multifocal of patches of centrilobular nodules and tree-in-bud in bilateral lungs.

Further diagnostic evaluation with computed tomography (CT) of the abdomen and lungs was conducted. The abdominal CT revealed mild peritoneal thickening with multiple miliary nodules and omental cake with ascites (Fig. 1a and b), while the lung CT demonstrated multifocal of patches of centrilobular nodules and tree-in-bud in bilateral lungs (Fig. 2a, b).

**Figure 3:**
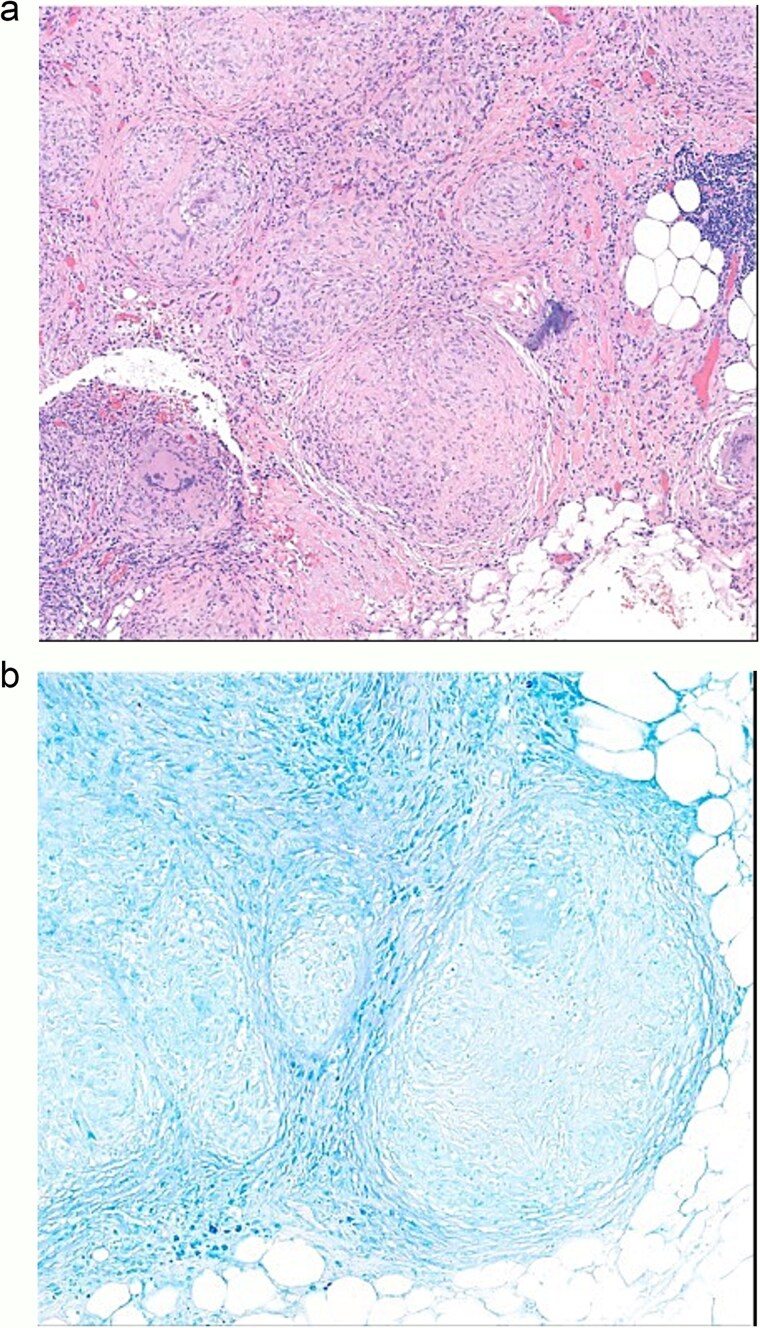
(a) Omentum pathology, non-necrotizing granulomatous inflammation. (b) Omentum pathology diagnosis, acid-fast stain negative.

Cytology examination showed no malignant cells. Subsequent pathological examination demonstrated non-necrotizing granulomatous inflammation (Fig. 3a). Special staining, including Periodic Acid-Schiff (PAS), Gomori Methenamine Silver (GMS), and acid-fast stains, was performed, but neither fungal elements nor acid-fast bacilli were identified (Fig. 3b).

We performed sonography-guided ascites tapping, and the adenosine deaminase (ADA) test was positive. Ascites culture yielded *Mycobacterium tuberculosis*. Under the impression of pulmonary TB with wet type TB peritonitis, the patient was initiated on anti-TB therapy with Akurit-4.

## Discussion

For 10 cases of abdominal TB associated with abdominal hernia [4–13], most cases revealed a thickened hernial sac with multiple small yellowish-white tubercles on the inner surface or omentum during surgical intervention. Additionally, straw-colored ascites was observed in most cases. In one case, pus was found at the hernia site during surgery, requiring perioperative intravenous antibiotics of cefuroxime and metronidazole [14]. For treatment, most cases involved the use of direct suture to close the defect, with only one case involving mesh insertion, and no postoperative infections were reported in the follow-up [7].

In herniorrhaphy, surgical meshes are the preferred choice for restoring the physical integrity and functional components of the musculofascial layers. However, between 1.4% and 6.9% of meshes require removal due to infection [15–17]. In patients with massive ascites, who are at a higher risk of infection, many surgeons remain hesitant to use mesh for hernia repair because of the increased risk of wound complications [18]. While hernia repair with mesh in patients with ascites, compared to suture repair, significantly reduces the recurrence rate, it is associated with an increased risk of complications such as infection, seroma, mesh erosion, and intestinal adhesion, obstruction, and fistula formation [19].

For peritonitis, treatment of empirical antibiotics includes a combination of antibiotics, such as second or third generation Cephalosporins (Cefuroxime/Ceftriaxone), plus Metronidazole or Piperacillin/Sulbactam [20].

When ascites was noted in surgery, we should consider different etiology, including heart failure, cirrhosis, kidney disease, malignant ascites, and infection peritonitis, such as TB or fungal infection. In our patient, he had no underlying heart disease or history of cancer. His renal and liver functions were within normal range. Besides, multiple white nodules were observed on the peritoneum. Initially, peritoneal metastasis or TB was considered; ultimately, peritoneal TB was confirmed.

## Conclusion

Massive ascites presents a significant challenge during hernioplasty, as mesh repair is generally avoided due to the elevated risk of infection. Peritoneal TB should be considered among the differential diagnoses in such cases.
